# Strategies for using nanoprobes to perceive and treat cancer activity: a review

**DOI:** 10.1186/s13036-016-0044-1

**Published:** 2017-03-23

**Authors:** Byunghoon Kang, Aastha Kukreja, Daesub Song, Yong-Min Huh, Seungjoo Haam

**Affiliations:** 1 Department of Chemical and Biomolecular Engineering, Yonsei University, 50 Yonsei-ro, Seoul, Korea; 2 Department of Radiology, Yonsei University, 50 Yonsei-ro, Seoul, Korea; 3 College of Pharmacy, Korea University, 2511 Sejong-ro, Sejong, Korea

**Keywords:** Nanoparticles, Stimuli responsive, Cancer activity

## Abstract

Nanomedicine has seen a significant increase in research on stimuli-responsive activatable nanoprobes for tumor-specific delivery and diagnosis. The tumor microenvironment has particular characteristics that can be exploited to implement therapeutic strategies based on disparities between normal tissues and tumor tissues, including differences in pH, oxygenation, enzymatic expression, gene activation/inactivation, and vasculature. The nanocarriers of activatable nanoparticles maintain their structure while circulating in the body and, upon reaching the tumor site, are altered by unique tumoral stimuli, leading to the release of a drug or other agent. This review demonstrates the latest achievements in the use of internal stimuli-responsive, activatable nanoparticles with respect to unique design strategies and applications.

## Background

Nanotechnology is a multidisciplinary research field offering exciting possibilities to revolutionize the field of biomedicine through transformative diagnostic and therapeutic tools [1–3]. The past decade has witnessed the successful introduction of a plethora of nanoparticles for cancer diagnosis, imaging, and treatment [4–7]. Nanoparticles have been fabricated with unique physical and chemical properties originating from myriad materials such as organic compounds, inorganic compounds, and hybrid compounds [2, 8, 9].

The early diagnosis of the pathological state of tumors is the mainstay of successful cancer treatment and personalized therapy [10]. Multifunctional nanoparticles, which provide both diagnostic and therapeutic features, have attracted great attention by providing early visualization of tumors and effective delivery of therapeutic agents with minimal side effects [11–15]. Nanoparticles engineered to carry a large payload of drug entities and target specific tumor sites represent an alternative to small-molecule imaging agents or drugs [5]. Targeting can be achieved through antibodies, aptamers, small tumor-specific peptides, polymers, and other molecules. Although targeting factors provide high cancer-cell specificity by binding to specific tumor epitopes, more efficient delivery systems are needed to control the release of the therapeutic cargo (drug, gene, or protein) from the nanocarrier. Such delivery systems must interact minimally with the biological components of the nanoparticle and must prevent the release of the cargo during circulation in the blood stream [16–19].

Irrespective of the presence of highly specific ligands, the heterogeneity of cancer-specific biomarkers among cancer types and organ sites makes it a challenging task to establish a foolproof strategy for cancer diagnosis [6, 15, 20]. To overcome that challenge, many recent studies have established the presence of biomarkers in the tumor microenvironment that are more consistent across a range of cancer types. The metabolism of cancer cells is very distinctive. Angiogenesis, dysregulated glycolysis leading to acidic pH and chronic oxidative stress, proliferative signaling, and the evasion of growth suppressors affecting enzymes and small molecules such as miRNA/DNA are all hallmarks of cancer. The targeting of those tumor hallmarks provides promising strategies for broad tumor detection [2, 15].

Activatable nanoparticles offer a platform to overcome the disadvantages of traditional tumor-targeting techniques by remaining intact before reaching the target. The on-demand activation of nanocarriers, which allows the efficient delivery of therapeutic agents with excellent dosage control, is becoming feasible. That approach requires the careful fabrication of nanoconstructs that are capable of undergoing specific, stimulus-induced changes such as conformational changes, hydrolytic cleavage, or specific protonation. Stimuli-responsive nanoconstructs can be transformed from a passive form to an active form in response to various exogenous or endogenous stimuli. Exogenous stimuli-responsive nanoconstructs take advantage of externally applied stimuli and include thermoresponsive systems, magnetic-responsive systems, ultrasound-triggered systems, light-triggered systems, and electroresponsive systems. Endogenous stimuli-responsive systems take advantage of the tumor microenvironment [15]. For example, at the cellular level, pH variations can be exploited to control drug release in late endosomes or lysosomes or in the generally low pH environment of cancer-specific sites. Also, the glutathione concentration varies between the extracellular environment and the intracellular environment and between tumor tissues and healthy tissues, potentially providing a way to attain redox sensitivity via the cleavage of disulfide bonds [21–23].

In this review, we discuss the most important progress made recently in nanoparticle synthesis. We also describe recent approaches that attempt to overcome the drawbacks of endogenous stimuli-responsive nanosystems for drug or gene delivery, with a particular emphasis on tumor treatment.

### Types of nanoparticles

#### Inorganic

**Fig. 1:**
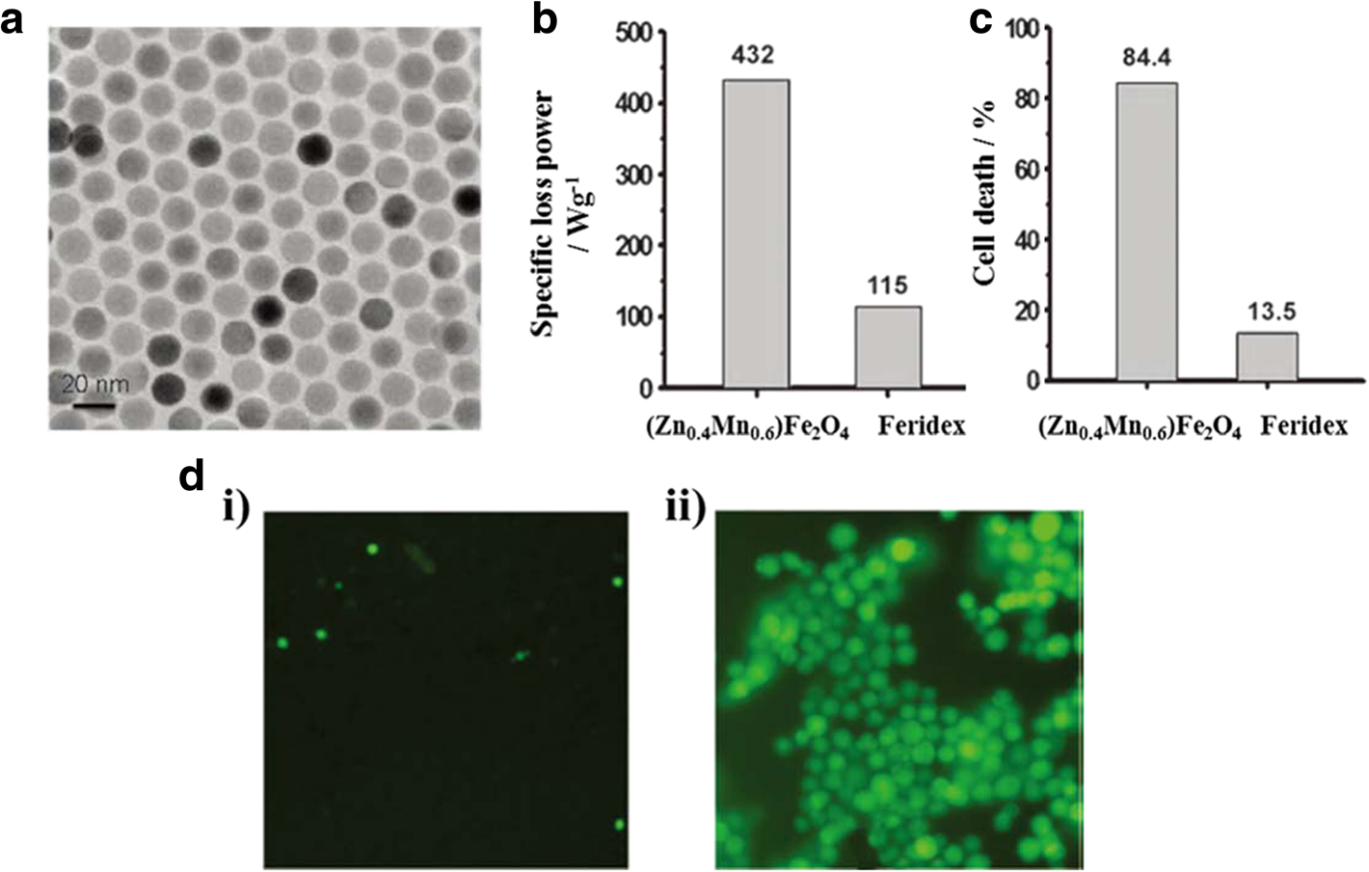
**a** TEM image of 15-nm (Zn_0.4_Fe_0.6_)Fe_2_O_4_ nanoparticles. **b** SLP values for (Zn_0.4_ Mn_0.6_)Fe_2_O_4_ and Feridex in a 500 kHz AC magnetic field with an amplitude of 3.7kAm^-1^. **c** Percentage of HeLa cells killed after treatment with (Zn_0.4_Mn_0.6_)Fe_2_O_4_ nanoparticles or Feridex and the subsequent application of an AC magnetic field for 10 min. **d** Fluorescence microscopy images of AC magnetic field applied HeLa cells treated with i) (Zn_0.4_Mn_0.6_)Fe_2_O_4_ nanoparticles or ii) Feridex. Calcein staining indicates live cells with green fluorescence. Reproduced with permission from ref. 27; Copyright 2009 Wiley-VCH

Table 1 shows the various types of nanoparticles currently used in biomedical research. Different classes of inorganic nanoparticles have recently gained much attention as potential diagnostic and therapeutic systems. Gold, iron oxide, silica, quantum dots, and other molecules have been investigated for the treatment and detection of diseases [24–30]. Super paramagnetic iron oxide nanoparticles (SPIONS) are probably the most explored nanoparticles for biomedical applications. Because of their innate magnetic properties, nanosized SPIONS are useful as contrast agents in magnetic resonance (MR) imaging for cancer diagnosis and as hyperthermia agents for cancer therapy. For example, Jang et al. [27] synthesized iron oxide nanoparticles with very high magnetism achieved by Zn^2+^ doping. Compared with conventional agents, the iron oxide nanoparticles provide eight to 14 times higher R2 values when used as MR contrast agents and four times greater specific loss power (SLP) values when used as hyperthermia agents (Fig. 1) [27]. Magnetic nanoparticles have also been utilized for cell separation, stem cell labeling, drug delivery, and magnetofection [31].

**Table 1:** Types of nanoparticles

Type		Examples	Reference
Inorganic	Metal Oxide	MFe_2_O_4_ (M = Fe, Mn, Co, Zn), Mn_x_O_y_ (1 ≤ x ≤ 3, 1 ≤ y ≤ 4), ZnO	Ref. [27, 31, 53]
	Noble Metal	Au, Ag, Pt	Ref. [32, 33]
	Quantum Dots	CdSe, ZnSe, ZnS, ZnO	Ref. [11]
	Silica	Mesoporous Silica, SiO_2_	Ref. [29, 36]
Organic	Polymer Based	Polymersome, Micelle	Ref. [37]
	Polysaccharide Based	Chitosan, Hyaluronic acid	Ref. [41]
	Lipid	Liposome, Cholesterol	Ref. [42]
	Peptide or Protein Nucleic Acid	siRNA, DNA	Ref. [43]
Hybrid	Inorganic/Organic Core/Shell		Ref. [44]
	Organic/Inorganic Core/Shell		Ref. [30]

Noble metal (gold, silver, and platinum) nanoparticles have unique surface plasmon resonance due to their nanoparticle-sized photon confinement [32]. Among them, gold nanoparticles are the most extensively studied, because their unique phonons make them advantageous for optical and photothermal applications. Several researchers have performed extensive studies to precisely control and tune the optical properties of gold nanostructures by changing the size, shape, and structure of the nanostructures [33–35].

Quantum dots are another example of inorganic nanoparticles that have emerged as versatile tools for biomedical imaging [11]. They are composed of atoms from groups II–VI or III–V of the periodic table. Quantum dots have unique optical and electrical properties due to quantum confinement effects. Recent studies have applied quantum dots in DNA hybridization, immunology, receptor-mediated endocytosis, in vitro and in vivo fluorescence imaging, multiplexed optical coding, and the high-throughput analysis of genes and proteins.

**Fig. 2:**
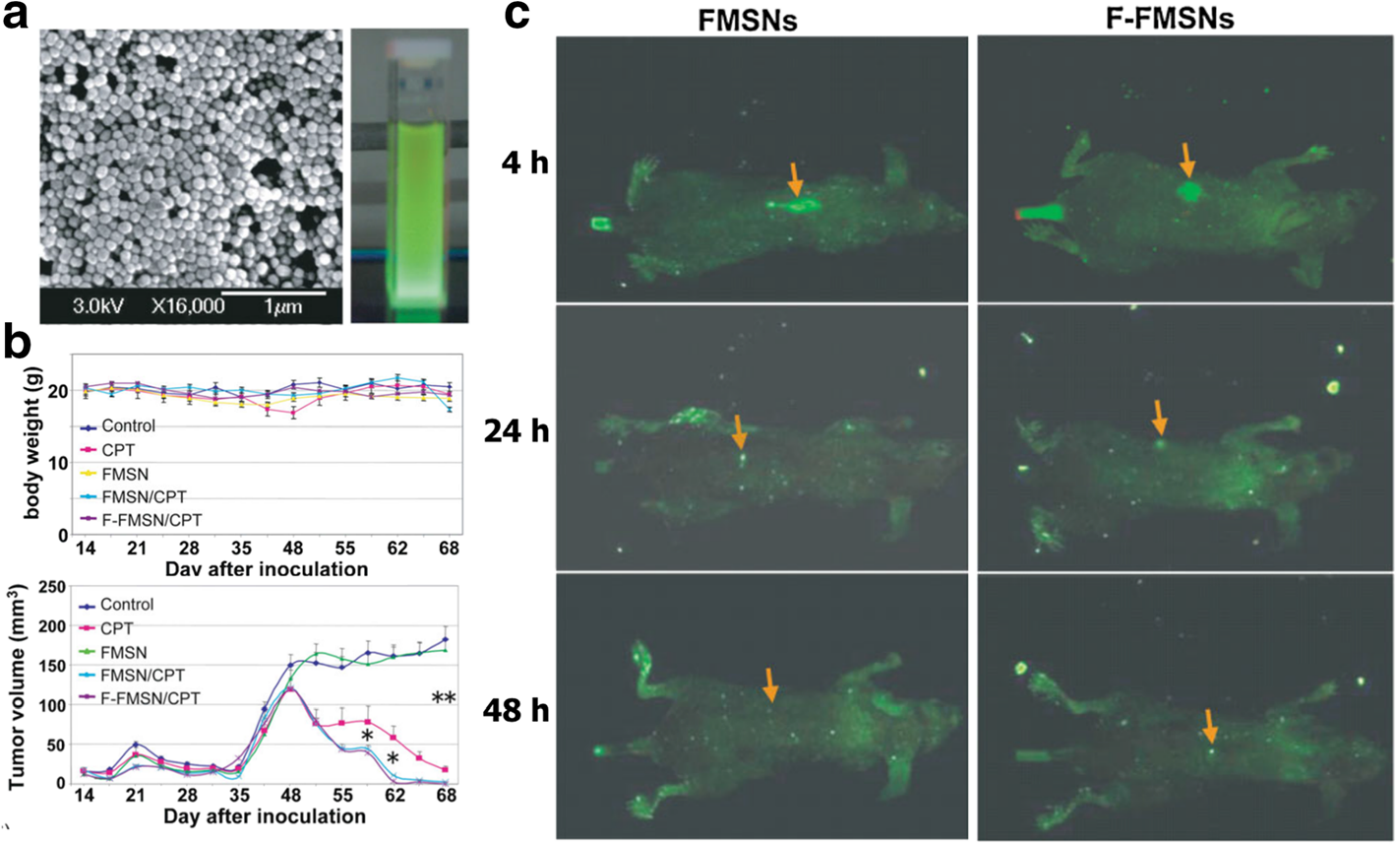
**a** Characterization of fluorescent mesoporous silica nanoparticles (FMSNs). SEM image of FMSNs and photograph showing bright-green photoluminescence from porous silicon nanoparticles under UV/Vis light. **b** Biodistribution of FMSNs in mice with xenograft tumors. Nude mice bearing subcutaneous human breast tumors were injected via the tail vein with FMSNs. Four hours later, the mice were anesthetized and analyzed with a Maestro 2 in vivo imaging system that produced green fluorescence images. The yellow arrows show the subcutaneous tumors. **c** Antitumor effects in mice of FMSNs loaded with Camptothecin (CPT). Animals were injected with either saline solution as a control, CPT, FMSNs without loading (FMSN), FMSNs loaded with CPT (FMSN/CPT), or F-FMSNs loaded with CPT (Folic acid-FMSN/CPT) twice per week until the end of the experiment (68 days). The average tumor volumes are shown as means ± SD. *p < 0.05; **p < 0.01. Reproduced with permission from ref. 29; Copyright 2010 Wiley-VCH

Silica nanoparticles are another important class of inorganic nanoparticles [29, 36]. Although silica nanoparticles do not have any special properties due to their sub-micrometer size, their structure can be tuned to control their size, shape, and porosity along with the presence of well-established siloxane chemistry for surface modifications. Those characteristics render silica nanoparticles suitable for diagnostic and therapeutic applications. Recently, Lu et al. [29] were able to suppress tumor growth in a human xenograft mouse model using mesoporous silica nanoparticles (MSNs) to deliver anticancer drugs [29]. In that work, the authors incorporated fluorescein isothiocyanate, an optical imaging agent, and the anticancer drug camptothecin (CPT) into the nanoparticles. As shown in Fig. 2, the CPT-loaded MSNs demonstrated excellent in vivo therapeutic efficacy in a xenograft model of MCF-7 human breast cancer cells.

### Organic nanoparticles

**Fig. 3:**
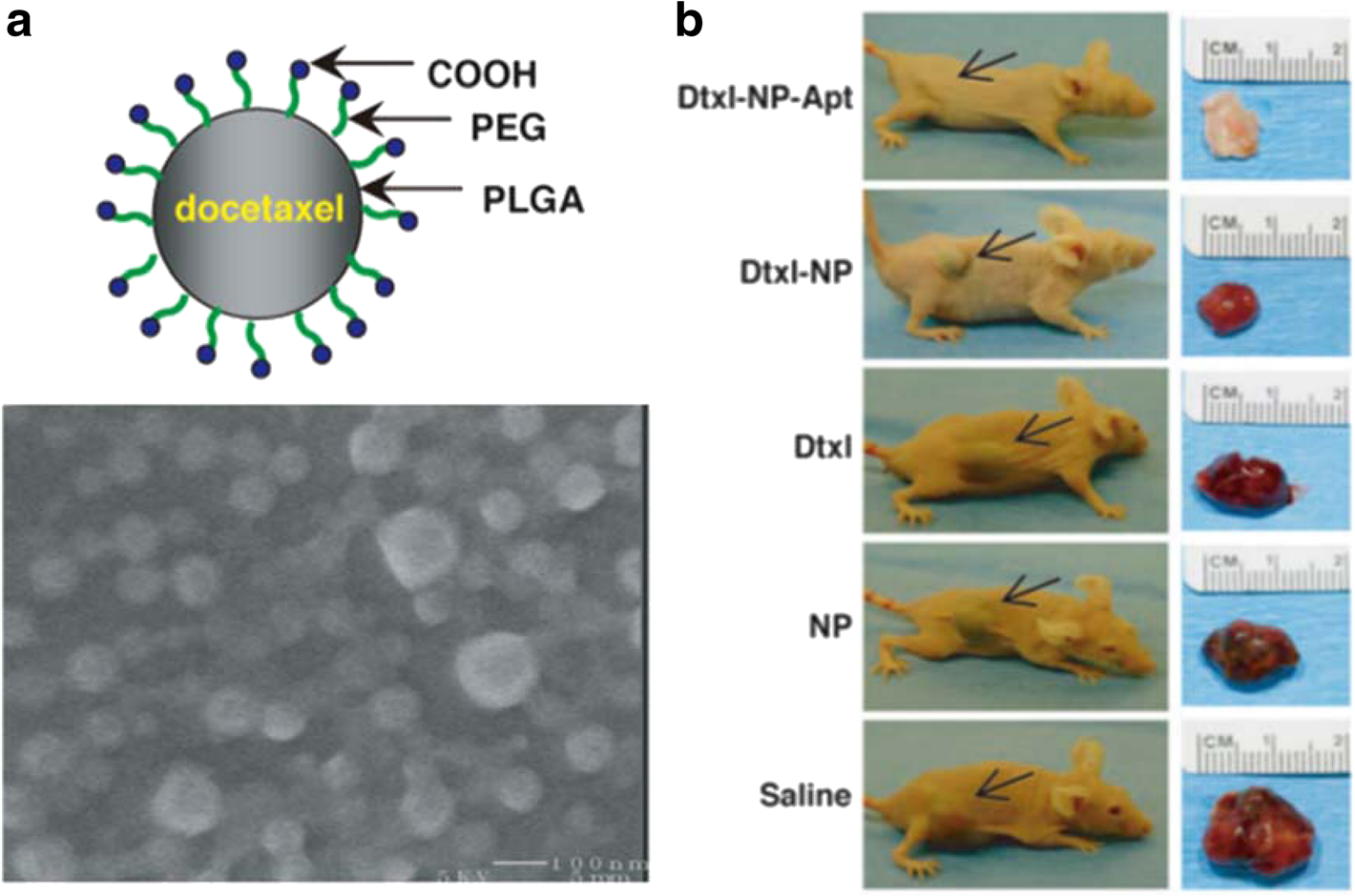
**a** Scheme and SEM image of Dtxl-encapsulated NPs. **b** The comparative efficacy study of single intratumoral injection (day 0) of saline, PEGylated PLGA NP without drug (NP), emulsified Dtxl (Dtxl), Dtxl-encapsulated NPs (Dtxl-NP), or Dtxl-encapsulated NP-Apt bioconjugates (Dtxl-NP-Apt). A representative mouse at the end point (>109 days) for each group is shown (*Left*) alongside images of excised tumors (*Right*). *Black arrows* indicate the position of the implanted tumor on each mouse. Reproduced with permission from ref. 40; Copyright 2006 PNAS

Organic nanoparticles are highly stable in biological fluids. They can be grouped into four major types: lipids, polysaccharides, peptides/proteins, and synthetic polymers. Polymeric nanoparticles are by far the most studied of the organic nanoparticles. A variety of synthetic polymers including polylactic acid (PLA), poly(lactic-co-glycolic) acid (PLGA), polyethylene glycol (PEG), and polyethyleneimine (PEI) have been utilized in nanoformulations that arrange them into polymer micelles, polymerosomes, polymer conjugates, or polymeric nanoparticles. Advancements in polymerization chemistry and careful control over targeting properties have enabled the engineering of multifunctional polymeric nanoparticles. For example, Farokhzad et al. [37] reported complete tumor reduction in a 109-day study in which animals were given a single intratumoral injection of docetaxel (dtxl)-encapsulated PLGA-b-PEG copolymer functionalized with RNA aptamers (Fig. 3) [38–40]. Some polymeric nanoparticles also exhibit electronic, opto-electronic, or photoluminescent behavior. Polyaniline, polypyrrole, polyacetylene, and their derivatives have been widely studied for their conductive properties, while polythiophenes, polyfluorenes, and poly (p-phenylene vinylene) have been explored for their electro-optical properties [37].

**Fig. 4:**
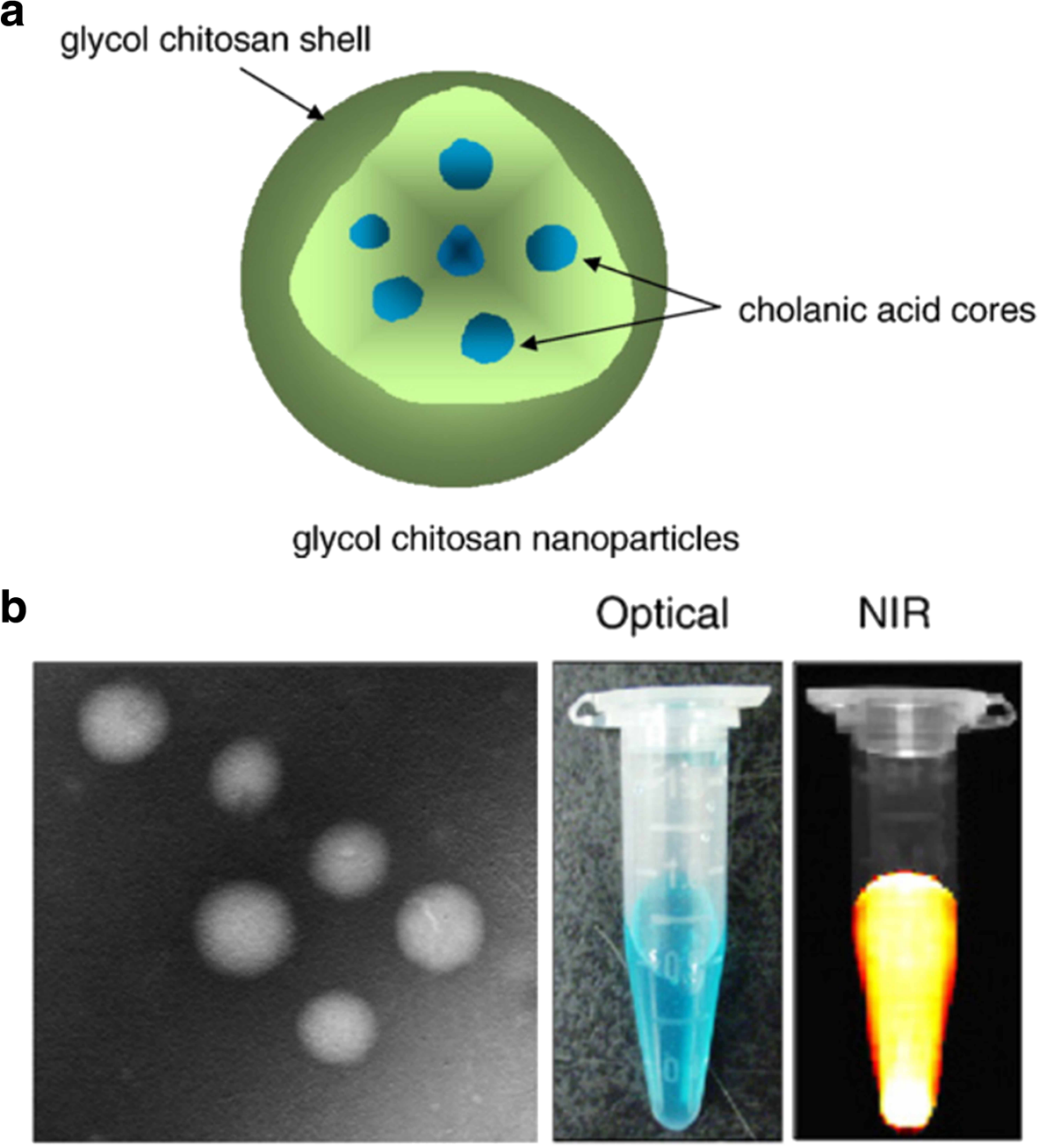
**a** Representative structure of glycol chitosan nanoparticles. **b** TEM, photograph, and near-infrared (NIR) fluorescence images of Cy5.5-labeled glycol chitosan nanoparticles. Reproduced with permission from ref. 41; Copyright 2007 ScienceDirect

Among the natural polymers, polysaccharides are the type most often studied for drug delivery. Chitosan nanoparticles are the polymers of choice for theragnostic applications. Park et al. [41] modified water-soluble glycol chitosan derivatives with cholanic acid to form tumor-targeting, chitosan-based nanoparticles. The high molecular weight chitosan nanoparticles showed enhanced tumor targeting due to their high in vivo stability (Fig. 4) [41].

Lipid-based nanocarriers play a major role in cancer therapy [42]. Nanocarriers, such as liposomes, lipid micelles, solid-lipid nanoparticles, nanosuspensions, and nanoemulsions, are commonly made of lipid-based materials, such as cholesterol, phosphatidylcholine, and 1,2-disteardyl-sn-glycero-3-phosphoethanolamine-N-[amino(polyethylene glycol)-2000] (DSPE-PEG 2000). Liposomes are the lipid-based nanoparticles that have been explored the most for cancer therapies. Liposomes are colloidal vesicles with single or multiple bilayered membrane structures. They are biodegradable and biocompatible and can encapsulate hydrophilic agents in their aqueous core and contain hydrophobic agents within their bilayers. Many clinical studies have shown the successful loading of hydrophobic drugs such as paclitaxel and doxorubicin into liposomes for cancer therapy [6].

A recent example of a protein-based nanosystem is Abraxane [43], which was approved by the FDA in 2005 and is now in clinical use. Abraxane is an albumin-bound paclitaxel nanoparticle produced in a high-pressure homogenizer. The drug particle is stabilized by human serum albumin and has an average size of 130 nm, which prevents any risk of capillary obstruction. Preclinical trials conducted in athymic mice with human breast cancer demonstrated that Abraxane has increased antitumor activity and greater penetration into tumor cells compared with an equal dose of standard paclitaxel. A phase I trial confirmed that the maximum tolerated dose of Abraxane is 70% higher than that of the Cremophor EL® paclitaxel formulation. A phase II trial confirmed that Abraxane has antitumor activity in patients with metastatic breast cancer. A phase III trial confirmed the superiority of Abraxane over standard paclitaxel in terms of both the overall response rate and the time to tumor progression.

### Hybrid nanocomposites

The development of hybrid nanocomposites, which combine organic and inorganic components, is intended to produce composite materials that retain the beneficial features of both organic compounds and inorganic compounds. Hybrid nanoparticles can be synthesized either by incorporating inorganic particles into a polymer matrix or by forming core/shell structures. Inorganic/organic core/shell structures combine a metal, semiconductor, metal oxide, or silica core with an organic/polymeric shell, which can save the metal core from oxidation and also increase biocompatibility. PEG, dextran, and chitosan have been studied extensively for the coating of various metal cores to improve biocompatibility and increase the number of applications for a single component. For example, Lim et al. [44] developed a multimodal nanoprobe using the amphiphilic polymer pyrenyl-PEG and superparamagnetic MnFe_2_O_4_ nanocrystals. The fluorescent magnetic nanoprobes were biocompatible and had excellent MR sensitivity and optical imaging capabilities [44].

Organic/inorganic core/shell nanoparticles have a polymer core and an inorganic shell. A metal oxide shell over a polymer can provide increased strength and abrasion resistance. Such systems are also used to synthesize inorganic, hollow nanoparticles [30].

### Types of stimulus response in the tumor microenvironment

The complexity and heterogeneity of cancer cells requires such cells to adapt and evolve aggressively, inducing the expression of key components of angiogenesis, hypoxia response, and glycolytic switching [15]. Those characteristics incite metabolic alterations that can change the pH [45, 46], miRNA and gene expression [47–51], and redox potential [52–61] of the tumor microenvironment. Table 2 shows the different types of internal stimuli present in the tumor microenvironment and the corresponding response materials to control the activation and behavior of nanostructures. Progress in the understanding of tumors at the molecular level and in the control of materials at the nanometer scale has allowed the development of new investigative tools for cancer therapy. For efficient cancer therapy, it is imperative that drugs or genes are delivered to the vicinity of the tumor without being degraded. Many researchers are developing engineered, stimuli-responsive, multifunctional nanoparticles that respond to changes in pH, redox potential, or enzyme activity for a variety of applications such as biomedical imaging, drug or gene delivery, and biosensing [6].

**Table 2:** Types of stimulus responses in the tumor microenvironment

Stimulus	Response materials	Reference
pH	poly(histidine-co-phenylalanine), 2-(diisopropyl amino) ethyl methacrylate	Ref. [45, 46]
Enzyme	MT1-MMP, MMP7, secretory phospholipase A2	Ref. [62, 63, 67]
Hypoxia and oxidative stress	4-nitrobenzyl group (hypoxic trigger), o-hydroxyl E-cinnamic ester (photo-activated group), MnO (glutathione), quaternized chlormethine (H_2_O_2_)	Ref. [52–61]
Nucleic acid based	molecular beacon	Ref. [47–51]

### pH

**Fig. 5:**
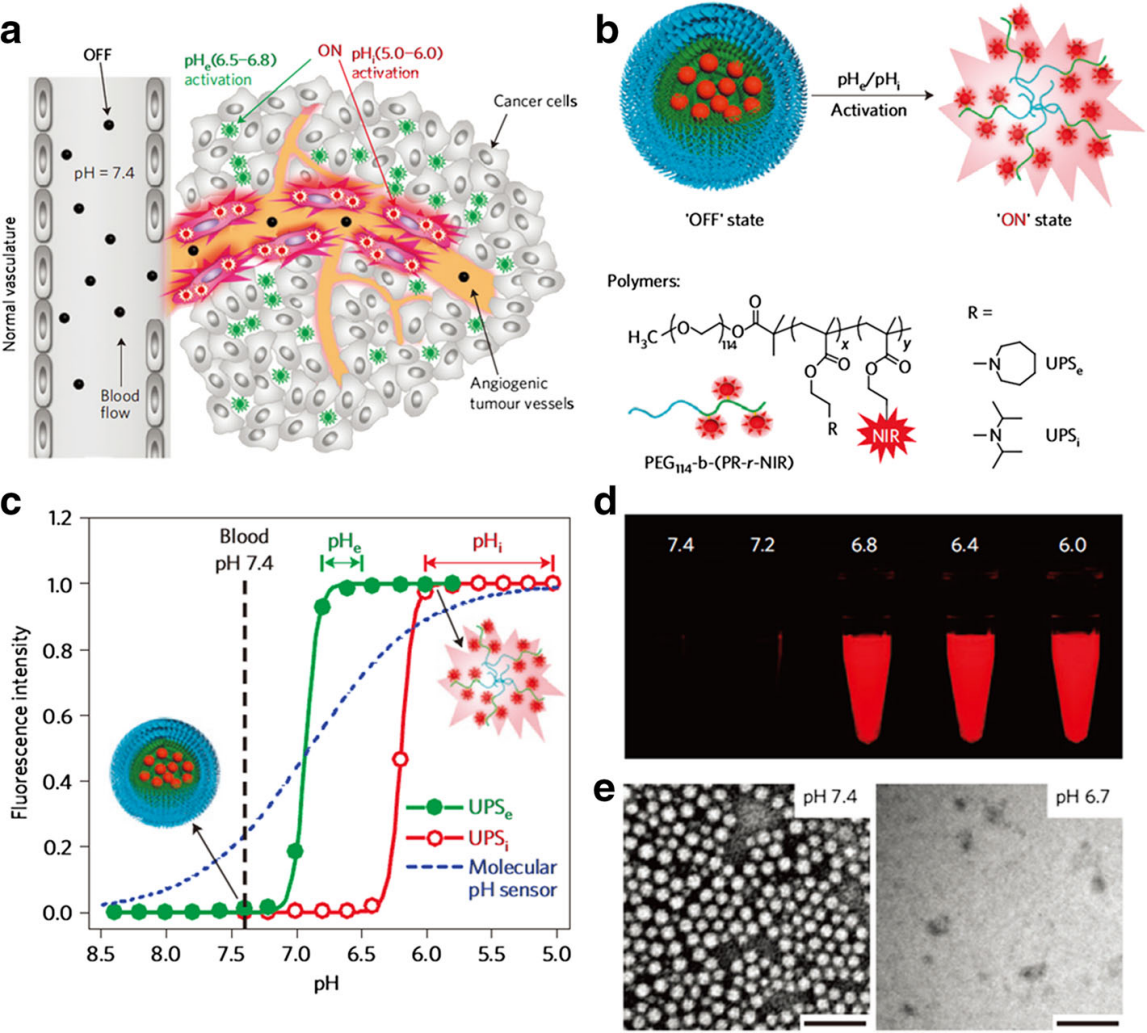
**a** Schematic of the imaging of the tumor microenvironment using ultra pH-sensitive (UPS) nanoprobes. The UPS nanoprobes stay ‘OFF’ at pH 7.4 during blood circulation. After reaching the tumor, the UPS nanoprobes are turned ‘ON’ by the acidic extracellular pH_e_ (6.5–6.8) in the tumor milieu or in endocytic organelles (pH_i_ 5.0–6.0) in the tumorous endothelial cells after receptor-mediated endocytosis. **b** Structural composition of the two types of nanoprobe, UPS_e_ and UPS_i_, with pH transitions at pH 6.9 and pH 6.2, respectively. **c** Normalized fluorescence intensity as a function of pH for UPS_e_ and UPS_i_ nanoprobes. At high pH (e.g., 7.4), both probes stay silent. At pH below the transition levels (i.e., pH 6.9 and 6.2), the nanoprobes can be activated as a result of micelle dissociation. **d** Fluorescent images of UPS_e_–Cy5.5 nanoprobe solution in different pH buffers (λ_ex_/ λ_em_ = 675/710 nm). **e** Transmission electron micrographs of UPS_e_ nanoprobes at pH 7.4 and pH 6.7 (polymer concentration = 1 mg/ml; scale bars = 100 nm). Reproduced with permission from ref. 46; Copyright 2014 Macmillan Publishers

Irrespective of the cancer type, the Warburg effect, or abnormally high rate of aerobic glycolysis, is a recognized hallmark of cancer and is perceived as a major biochemical alteration associated with malignant transformation [21, 22]. Otto H. Warburg observed that liver cancer cells display increased glycolytic activity in the presence of oxygen compared with normal cells. Instead of using pyruvate, cancer cells ferment glucose into lactic acid to generate ATP, even in the presence of oxygen. Unlike normal cells, cancer cells produce approximately 60% of their ATP from glycolysis. Therefore, cancer cells and tissues have an acidic pH. Various researchers have used that metabolic difference between normal cells and cancer cells as a biochemical basis to develop anticancer therapeutic and imaging strategies [23]. Wang et al. [46] demonstrated a signal amplification strategy using ultra pH-sensitive (UPS) fluorescent nanoprobes [46]. UPS nanoparticles are composed of three components: a UPS core that provides a sharp pH transition (at pH 6.5 ~ 6.8); fluorophores that provide homo-FRET quenching; and a Arg-Gly-Asp that provides a targeting moiety. As shown in Fig. 5, UPS nanoparticles activate strongly upon suitable changes in the physiological pH, amplifying the signal of the tumor microenvironment. The endosomal/lysosomal pH, which is 1.4–2.4 units lower than the physiological pH, can be exploited by pH-sensitive nanocarriers encapsulating drugs, genes, or contrast agents. Such nanocarriers bind to the endosomal membrane after endocytosis and eventually release their payload into the cytoplasm when they become destabilized by the low pH. For example, Kim et al. [45] designed an endosomal pH-triggered drug-delivery system [45]. The objective was to avoid premature drug release in the extracellular environment and also to avoid toxicity due to the leakage of digestive lysosomal enzymes. They used a mixed micelle approach to design a doxorubicin-loaded micelle composed of a pH-sensitive core [poly(histidine (His)-*co*-phenylalanine (Phe))-*b*-polyPEG)], which is stable at pH 7 but unstable at pH 6.5, and another destabilization pH-adjusting blending polymer [poly(L-lactic acid) (PLLA)-*b*-PEG-folate], which causes destabilization at pH 6. The activation at endosomal pH combined with folate receptor-mediated endocytosis effectively accelerated doxorubicin release at pH 6.0, resulting in the efficient killing of cancer cells in vitro. Choi et al. demonstrated another application of a pH-based system in their investigation of the endosomal-lysosomal system. They developed a polyaniline (PANI)-based organic quencher for intracellular compartmental trafficking by adsorbing multi core-shell Fe3O4/MnO/Silica/PANI nanoparticles with Cy3 and Cy7 fluorophores to efficiently quench the emeraldine-base and emeraldine-salt forms of PANI, respectively. Changes in the pH throughout the endosomal-lysosomal pathway led to reversals in the transition states of the emeraldine base and the emeraldine salt, quenching the Cy3 emission and activating the Cy7 emission.

### Enzyme

Enzymes are key components in all biological processes. The dysregulation of enzyme activity has been observed in many pathological conditions, rendering the detection of enzyme expression a powerful tool for diagnosis [62, 63]. The exceptional efficiency of enzymes in the selective recognition of their substrates makes them a sophisticated tool for producing biologically inspired chemical reactions. That has led to a growing interest in the development of bioresponsive nanoparticle systems, including polymeric nanoparticles, liposomes, metal and semiconducting nanoparticles, and silica nanoparticles, that respond to the catalytic activity of enzymes. Nanoparticles can be rendered enzyme-responsive by the inclusion of moieties that can either be cleaved upon recognition by a biocatalyst or be transformed upon catalytic action by an enzyme [64]. Cancer-associated proteases, esterases, phospholipases, and oxidoreductases are upregulated in tumors and have been utilized to develop enzyme-responsive nanosystems. For example, phospholipase A2 is upregulated in various tumors, including those of the prostate. Enzyme-responsive nanosystems have been explored recently in the search for activatable liposomal drug-delivery systems. Linderoth et al. [65] developed a novel drug-delivery system that combines lipid-based prodrugs formulated as liposomes with overexpressed secretory phospholipase A2 (sPLA2) as a trigger for activation [65]. As a model drug, they used capsaicin prodrug 8, which forms a uniform bilayer of vesicles directly upon dispersion in a buffer. The ester group at the Sn^-2^ position of glycerophospholipids is hydrolyzed by sPLA2 , so the researchers synthesized a glycerophospholipid derivative with the drug at the Sn^-1^ position. When the ester group at the Sn^-2^ position was hydrolyzed, an OH group was released, which reacted with the ester group at the Sn^-1^ position to form a lactone and thereby release the drug.

**Fig. 6:**
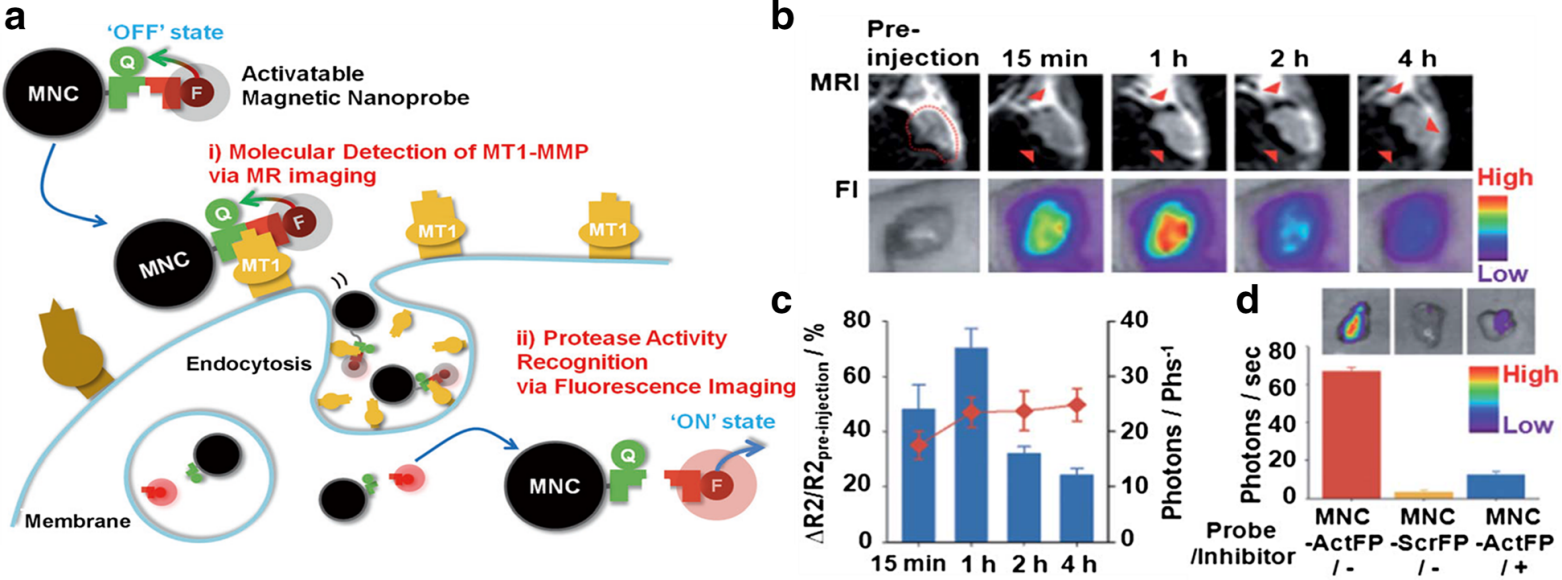
**a** The dual imaging process of activatable magnetic nanoprobes (magnetic nanocrystals conjugated with activatable fluorogenic peptides, MNC-ActFP) for i) molecular detection of MT1-MMP anchored on invasive cancer cells by MR imaging and ii) sensitive recognition of the proteolytic activity of MT1-MMP by fluorescence imaging. Q = quencher, F = fluorescence dye. **b** In vivo MR (*Upper*) and NIR fluorescence (*Lower*) images of tumor-bearing mice at different time points after intravenous injection of MNC-ActFP. Red arrowheads in the MR images indicate the signal-enhanced sites of the tumor. **c** ΔR2/R2 nontreatment (line scatter) and NIR fluorescence intensity (bar graphs). **d** NIR fluorescence images (upper) and their intensity graph (lower) for excised tumors from tumor-bearing mice 1 h after intravenous injection of MNC-ActFP, MNC-ScrFP, or MNC-ActFP plus inhibitor. Reproduced with permission from ref. 67; Copyright 2011 Wiley-VCH

Proteases are the most commonly exploited enzymes. Matrix-metalloproteases (MMPs) play an essential role in tumor-cell invasion into connective tissue [66]. Researchers have utilized that role to detect invasive cancer cells by comparing the MMPs of malignant cells with the secreted, soluble MMPs of normal cells. Park et al. [67] developed an enzyme-activatable, bimodal imaging probe for the simultaneous determination of the expression and proteolytic activity of the MT1-MMP present on the surface of invasive cancer cells. They used an activatable fluorogenic peptide (ActFP) that acts as both a targeting moiety and a proteolytic site for MT1-MMP [67]. ActFPs provide fluorogenic activity by combining an NIR dye with a quencher to induce a FRET effect. Upon enzymatic cleavage of the MMP ligand, the NIR dye fluoresces in the cytoplasm. ActFP probes can also be conjugated with magnetic nanoparticles for combined fluorescence and MR imaging (Fig. 6).

Polymeric nanoparticles are the most widely used nanoplatforms for the development of enzyme-responsive systems. [66] Chemotherapeutic drugs, proteins, genes, and siRNAs have been delivered using enzyme-responsive polymeric nanoparticles. In a recent study, Li et al. [66] developed a smart polymeric nanoparticle containing a positively charged dimethylaminoethyl (DMAEMA) corona to package siRNA and also act as a pH-responsive core. They linked the corona to a PEG layer via an MMP-7 cleavage peptide, which shielded the nanoparticles from nonspecific cell interactions. Once the PEG layer was cleaved in an MMP-7-rich environment, the nanoparticles became positively charged, and their rate of internalization increased 2.5-fold because of the negative-positive charge interactions. The pH change following internalization further disrupted the corona, leading to siRNA escape from the endolysosomal pathways.

### Hypoxia and oxidative stress

Tumors have inadequate vasculature and therefore rapidly exhaust their blood supply, leading to glucose deprivation and hypoxia. Glucose deprivation prevents the decomposition of endogenous oxygen radicals, causing oxidative stress. Hypoxia and oxidative stress are both present in tumor cells and are interlinked. Angiogenesis within the tumor tissue causes periods of hypoxia due to the uncontrolled blood flow. Hypoxia is known to promote aggressive tumor phenotypes and causes resistance to chemotherapy and radiotherapy. Recently, many studies have applied a hypoxia-activated strategy to release prodrugs, imaging agents, and other functional molecules within tumor cells. Feng at al. [60] developed a modified gemcitabine (GMC)-based pro-prodrug (GMC-CA_E_-NO2) with an o-hydroxyl E-cinnamic ester photo-activated group (CA_E_) and a nitro-benzyl group, which could not be reduced under normal oxygen conditions [61]. Under hypoxic conditions, the GMC-CA_E_-NO_2_ was converted to the prodrug GMC-CA_E_. Subsequently upon UV exposure, alteration of the prodrug led to the formation of fluorescent dye and GMC release. The rate of GMC release increased with decreasing O_2_ concentration.

Reactive oxygen species (ROS); including hydrogen peroxide (H_2_O_2_), hypochlorous acid (HOCl), superoxide (O_2_^-^), singlet oxygen (^1^O_2_), and hydroxyl radical (OH^-^); are abundant in cancer cells. The overproduction of ROS leads to redox imbalance and cellular damage. Li et al. [55] developed chlormethine (Chl), an H_2_O_2_-sensitive quaternized prodrug with an eight-member cyclic boronate ester that could be triggered in the presence of H_2_O_2_ [55]. They covalently linked the prodrug to poly(fluorene-co-phenylene; PFP) side chains, creating PFP-Chl, which successfully released the Chl within cancer cells and inhibited cell growth. In another study, Chen at al. [58] developed H_2_O_2_-activatable and O_2_-evolving photodynamic therapy (PDT) nanoparticles (HAOP NPs). They encapsulated methylene blue (photosensitizer) and catalase (O_2_-evolving agent) in the aqueous core of a PLGA shell and doped the bilayer of the shell with black-hole quencher-3 (BHQ-3). They further modified the surface of the particles with c(RGDFK), a tumor-targeting peptide. The HAOP NPs were selectively taken up by αvβ3 integrin-rich tumor cells, followed by H_2_O_2_ penetration into the core, catalysis by catalase, and O_2_ generation, causing the rupture of the polymer shell. Thus, the nanoparticles allowed the controlled release of ^1^O_2_ within tumor cells, providing high-efficiency in vivo PDT while also overcoming hypoxia-induced drug resistance.

**Fig. 7:**
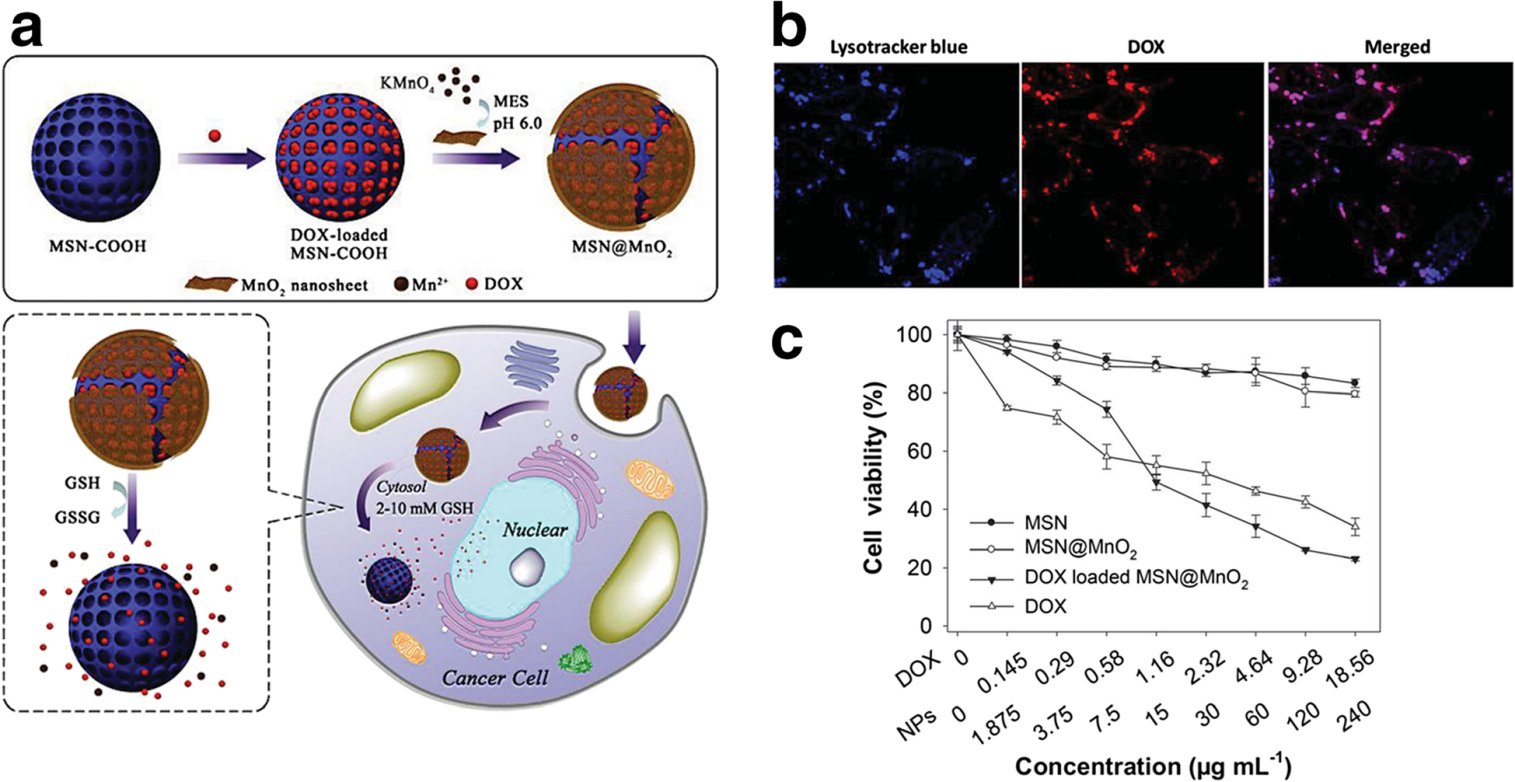
**a** Schematic illustration of the formation of DOX-loaded MSN@MnO_2_ and GSH-triggered drug release in cancer cells. **b** Fluorescence images of HepG2 cells incubated with DOX-loaded MSN@MnO_2_ for 3 h. **c** Viability of HepG2 cells after being treated by free DOX, MSN, MSN@MnO_2_, or DOX-loaded MSN@MnO_2_ for 48 h. NPs = nanoparticles. Reproduced with permission from ref. 53; Copyright 2015 Wiley-VCH

Glutathione (GSH) plays a major role in protecting cells against oxidative stress by undergoing a conversion from reduced GSH to oxidized GSSG [53]. The concentration of GSH is extremely high in tumor cells, making it an ideal biomarker. Yang et al. [53] synthesized GSH-responsive, MSHs for controlled drug release. As shown in Fig. 7, they reduced KMnO_4_ to generate an MnO_2_ nanolayer over MSHs loaded with doxorubicin. In the presence of GSH, the MnO_2_ nanostructure dissociated, leading to the formation of Mn^2+^ and the release of doxorubicin through the mesopores. In other research, Kim et al. developed activatable T1 and T2 dual-mode MR imaging agents to avoid MR contrast enhancement upon nonspecific interactions. They synthesized an Fe_3_O_4_ core/Mn_3_O_4_ shell nanosystem in which the Mn_3_O_4_ shell shielded the iron oxide against water protons and thus inhibited T2 contrast enhancement. The Mn_3_O_4_ shell also acted as a redox switch that activates in the presence of glutathione, releasing Mn^3+^ ions (to provide T1 contrast enhancement) and allowing the iron oxide core to interact with water protons (to provide T2 contrast enhancement). The researchers demonstrated effective passive tumor targeting for T1 and T2 weighted MR imaging in a xenograft tumor model.

### Nucleic acids

**Fig. 8:**
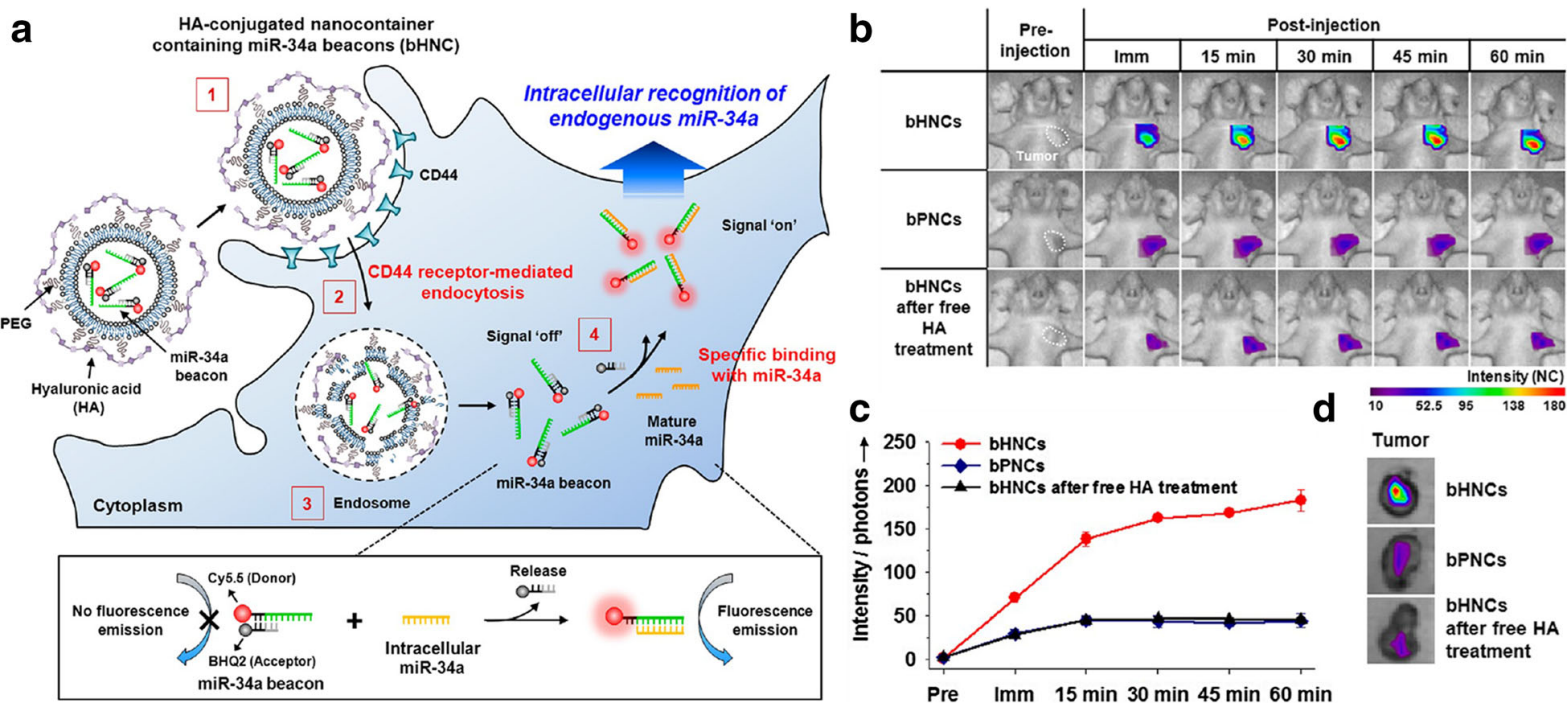
**a** Schematic illustration of the miR-34a beacon delivery system for targeted intracellular recognition of miR-34a based on HA-coated nanocontainers that encapsulate the miR-34a beacons (bHNCs); consecutive processes of (*i*) binding to CD44 receptors, (*ii*) internalization into an endosome, (*iii*) disassembly of bHNCs leading to the destabilization of endosome membranes after pH reduction, and (*iv*) final displacement of the miR-34a beacons from the HNCs permitting transport into the cytoplasm. **b** In vivo and ex vivo imaging of miR-34a in an orthotopic breast cancer model. In vivo optical fluorescence images of MDA-MB-231 tumor-bearing mice after the intravenous injection of bHNCs, bPNCs, and bHNCs after free HA treatment (10 mg/mL of HA in PBS per animal) at various time intervals. Tumor regions are indicated by a white dashed boundary. **c** Total photon counts in tumor regions after injection of bHNCs, bPNCs, and bHNCs after free HA treatment (miR-34a beacon concentration = 5 nmol). **d** Ex vivo optical fluorescence images of tumors excised 1-h post injection of bHNCs, bPNCs, and bHNCs after free HA treatment. The intensity maps on the fluorescence images are displayed as the normalized photon counts (NC) per point with laser power (5.0 μW) and integration time (0.4 s). Reproduced with permission from ref. 42; Copyright 2012 ACS Publications

Abnormalities in gene expression cause cancer, providing tumor cells with essential alterations in aspects of cell physiology such as apoptosis, metastatic potential, angiogenesis, and growth/anti-growth signaling [18]. Increased understanding of gene expression and the development of techniques to detect expression levels have led to new means of early cancer diagnosis and therapy. During the past decade, increased attention has been given to DNA, mRNA, and miRNA, which are present at high concentrations in patients with cancer compared with healthy individuals. Abnormally expressed miRNAs have been increasingly utilized because of their fundamental role in cancer metastasis [51]. Well-tailored, activatable nanostructures for tumor detection and suppression have been designed to deliver oligonucleotides to cells without being degraded by endogenous nucleases. For example, Kim et al. [42] reported a hyaluronic-based nanocontainer with miR-34a beacons that could be used to detect breast cancer [42]. Upon injection, the nanocontainers bind to CD44 receptor and become internalized by endosomes, where they are subsequently disrupted by the low pH, leading to the release of miR-34a beacons into the cytoplasm. The miR-34a beacons contain a linear oligonucleotide that is complementary to miR-34a. That oligonucleotide is conjugated to Cy5.5 dye and is also annealed to a shorter oligonucleotide that is conjugated to black-hole quencher 2 (BHQ-2) as a fluorescent acceptor. When the beacons are released into the cytoplasm (Fig. 8), miR-34a binds to the complementary sequence, displacing the BHQ-2 and thus turning on the signal. The researchers successfully detected and created images of miR-34a in breast cancer animal models. Shi et al. [68] utilized cell-membrane protein kinase-7 (PTK7) to activate an aptamer probe [68]. The activatable aptamer probe (AAP) consisted of three fragments: a cancer-targeted aptamer sequence (A-strand), a poly T linker (T-strand), and a short DNA sequence (C-strand) complementary to a part of the A-strand with a fluorophore and quencher attached to both termini. The hybridization of the A-strand to the C-strand resulted in a hairpin conformation that holds the fluorophore and quencher together, keeping the nanostructure in a quenched state. When the AAP binds the cell-membrane protein receptor, spontaneous conformational reorganization occurs, separating the fluorophore from the quencher and switching the signal on. The AAPs successfully displayed enhanced contrast in vitro and in vivo compared with ‘always-on’ probes, facilitating sensitive detection of early-stage cancer.

Cancer-specific mRNAs have been utilized to detect tumor progression. Li et al. [49] utilized multiple mRNA targets to improve the accuracy of cancer detection in single-marker assays [49]. They synthesized a multicolor fluorescent nanoprobe consisting of gold nanoparticles functionalized with three short, dye-terminated reporter sequences via a gold-thiol linkage. The gold nanoparticles quench the fluorescence of the dye. Upon RNA or DNA hybridization with a more stable complementary sequence, the reporter sequence is released, turning the fluorescent signal on. The researchers successfully distinguished between cancer cells and normal cells and reported changes in the expression levels of tumor-related mRNAs.

### Limitations of stimuli-responsive nanoparticles

Stimuli-responsive nanosystems have seen tremendous growth in the past few decades. Their efficacy for cancer detection and therapy is undeniable; however, certain challenges still exist that need to be addressed and may vary from one patient to another. The pH-activated nanosystems that disrupt the lysosomal membrane may lead to the release of lysosomal enzymes into the cell cytoplasm, which can cause autophagy and cell death. Also, the release of payload inside the lysosomes may lead to denaturation, causing significant loss of efficacy. Enzyme-activated systems also face various challenges. Enzyme dysregulation in various diseases and at various stages of the same disease needs to be studied extensively for eventual clinical translation. Overlapping substrates between closely related enzymes can cause nonspecific uptake or cleavage, resulting in systemic toxicity. Nucleic acid-activated nanoparticles have a major drawback based on the fact that protein upregulation is not always related to nucleic acid upregulation in cancer, which may lead to untrustworthy conclusions. Hypoxia and oxidative stress is present at elevated levels in all cancer types. However, high levels of ROS may cause activation of various signaling pathways leading to cell death.

## Conclusions and perspectives

We reported a general overview of the role of nanoparticles in the efficient delivery of drugs, genes, contrast agents, and other functional molecules for cancer imaging and therapy via specific targeting and selective activation in the cellular niche. Tumor microenvironment-activatable nanosystems with nanocarriers acting as ‘homing devices’ loaded with therapeutic contrast/therapeutic agent and coated with responsive polymers or probes provide new insights into cancer therapy by demonstrating high specificity and sensitivity with minimal degradation or background signal. The successful translation of activatable nanosystems into clinical trials will change the very foundation of tumor theragnostics. Their controlled release, specific targeting, and biocompatibility will make them an important component of personalized therapy in the near future. Nevertheless, some limitations still exist, and more data is needed to translate the results obtained in animal models into applications in humans. The experimental models in humans are not yet standardized and much more heterogeneous than animal models because of high heterogeneity in blood flow, which often makes comparison of results troublesome. In vivo systems are complex, and studies and regulations are essential to ensure the biocompatibility of nanocarriers in humans. We anticipate that many of the current problems will be resolved in the near future, and we expect that much of the current research will be translated into clinical applications.

## References

[1] Caruso F, Hyeon T, Rotello VM (2012). Chem Soc Rev.

[2] Davis ME, Chen ZG, Shin DM (2008). Nat Rev Drug Discov.

[3] Yin Y, Talapin D (2013). Chem Soc Rev.

[4] Sampath CA, Arun KI, Mansoor A (2011). Accounts of chemical research.

[5] Lee DE, Koo H, Sun IC, Ryu JH, Kim K, Kwon IC (2012). Chem Soc Rev.

[6] Petros RA, DeSimone JM (2010). Nat Rev Drug Discov.

[7] Kamaly N, Xiao Z, Valencia PM, Radovic-Moreno AF, Farokhzad OC (2012). Chem Soc Rev.

[8] Zaera F (2013). Chem Soc Rev.

[9] Torchilin VP (2014). Nat Rev Drug Discov.

[10] Park K, Lee S, Kang E, Kim K, Choi K, Kwon IC (2009). Advanced Functional Materials.

[11] Lee S, Park K, Kim K, Choi K, Kwon IC. Chem Commun (Camb). 2008;28:4250–4260. 10.1039/b806854m 18802536

[12] Jonathan F. Lovell, T. W. B. L., Juan Chen, Gang Zheng. Chemical Reviews 2010, 110. 10.1021/cr900236h 20104890

[13] Coti KK, Belowich ME, Liong M, Ambrogio MW, Lau YA, Khatib HA, Zink JI, Khashab NM, Stoddart JF (2009). Nanoscale.

[14] Hisataka kobayashi, P. L. C. Accounts of chemical research 2011, 44, 83–90. 10.1021/ar1000633 PMC3040277 21062101

[15] Danhier F, Feron O, Preat V (2010). J Control Release.

[16] Zhang G, Surwade SP, Zhou F, Liu H (2013). Chem Soc Rev.

[17] Kontos S, Hubbell JA (2012). Chem Soc Rev.

[18] Kanjiro Miyata NN, Kazunori K (2012). Chemical Society Reviews.

[19] Palivan CG, Fischer-Onaca O, Delcea M, Itel F, Meier W (2012). Chem Soc Rev.

[20] Rajendran L, Knolker HJ, Simons K (2010). Nat Rev Drug Discov.

[21] Douglas Hanahan RAW (2000). Cell.

[22] Vander MG, Heiden LCC, Craig B (2009). Thompson. SCIENCE.

[23] Chen Z, Lu W, Garcia-Prieto C, Huang P (2007). J Bioenerg Biomembr.

[24] Huanjun Chen XK, Zhi Y, Weihai N, Jianfang W (2008). Langmuir.

[25] Xu ZP, Zeng QH, Lu GQ, Yu AB (2006). Chemical Engineering Science.

[26] Huang HC, Barua S, Sharma G, Dey SK, Rege K (2011). J Control Release.

[27] Jang JT, Nah H, Lee JH, Moon SH, Kim MG, Cheon J (2009). Angew Chem Int Ed Engl.

[28] Brian L. Cushing VLK, Charles J. O’Connor. Chemical Reviews. 2004;104:3893–3946. 10.1021/cr030027b 15352782

[29] Lu J, Liong M, Li Z, Zink JI, Tamanoi F (2010). Small.

[30] Kango S, Kalia S, Celli A, Njuguna J, Habibi Y, Kumar R (2013). Progress in Polymer Science.

[31] Pan Y, Du X, Zhao F, Xu B (2012). Chem Soc Rev.

[32] Arvizo RR, Bhattacharyya S, Kudgus RA, Giri K, Bhattacharya R, Mukherjee P (2012). Chem Soc Rev.

[33] Jans H, Huo Q (2012). Chem Soc Rev.

[34] Dreaden EC, Alkilany AM, Huang X, Murphy CJ, El-Sayed MA (2012). Chem Soc Rev.

[35] Chen H, Shao L, Li Q, Wang J (2013). Chem Soc Rev.

[36] Vivero-Escoto JL, Huxford-Phillips RC, Lin W (2012). Chem Soc Rev.

[37] Farokhzad OC, Cheng J, Teply BA, Sherifi I, Jon S, Kantoff PW, Richie JP, Langer R (2006). Proc Natl Acad Sci U S A.

[38] Brayner R, et al. (eds.) Nanomaterials: A Danger or a Promise?, In :Synthesis of Organic and Bioorganic Nanoparticles: An Overview of the Preparation Methods. Springer-Verlag Allouche J. 2013;27–74.

[39] Hong-Bing Fu J-NY (2001). J American Chemical Society.

[40] Yhee JY, Son S, Kim N, Choi K, Kwon IC (2014). MRS Bulletin.

[41] Park K, Kim JH, Nam YS, Lee S, Nam HY, Kim K, Park JH, Kim IS, Choi K, Kim SY, Kwon IC (2007). J Control Release.

[42] Eunjung Kim JY, Joseph P, Soonhag K, Nam Hee K, Jong In Y, Jin-Suck S, Seungjoo H, Yong-Min H (2012). ACS Nano.

[43] Zhao M, Lei C, Yang Y, Bu X, Ma H, Gong H, Liu J, Fang X, Hu Z, Fang Q (2015). PLoS One.

[44] Lim EK, Yang J, Dinney CP, Suh JS, Huh YM, Haam S (2010). Biomaterials.

[45] Kim D, Lee ES, Oh KT, Gao ZG, Bae YH (2008). Small.

[46] Wang Y, Zhou K, Huang G, Hensley C, Huang X, Ma X, Zhao T, Sumer BD, DeBerardinis RJ, Gao J (2014). Nat Mater.

[47] Schwarzenbach H, Hoon DS, Pantel K (2011). Nat Rev Cancer.

[48] Kim E, Lee H, An Y, Jang E, Lim E-K, Kang B, Suh J-S, Huh Y-M, Haam SJ (2014). Mater. Chem. B.

[49] Li N, Chang C, Pan W, Tang B (2012). Angew Chem Int Ed Engl.

[50] Dwight S. Seferos, D. A. G., Haley D. Hill, Andrew E. Prigodich, Chad A. Mirkin. J. Am. Chem. Soc. 2007, 129, 15477-15479. 10.1021/ja0776529 PMC3200543 18034495

[51] Xiang-Hong Peng Z-HC, Jin-Tang X, Carlson GW, Lewis MM, Wood WC, Lily Y (2005). Cancer Res.

[52] Kong F, Liang Z, Luan D, Liu X, Xu K, Tang B (2016). Anal Chem.

[53] Yang X, He D, He X, Wang K, Zou Z, Li X, Shi H, Luo J, Yang X (2015). Particle & Particle Systems Characterization.

[54] Kim EJ, Bhuniya S, Lee H, Kim HM, Cheong C, Maiti S, Hong KS, Kim JS (2014). J Am Chem Soc.

[55] Li M, Li S, Chen H, Hu R, Liu L, Lv F, Wang S (2016). ACS Appl Mater Interfaces.

[56] Lee D, Park S, Bae S, Jeong D, Park M, Kang C, Yoo W, Samad MA, Ke Q, Khang G, Kang PM (2015). Sci Rep.

[57] Qiu FY, Zhang M, Ji R, Du FS, Li ZC (2015). Macromol Rapid Commun.

[58] Chen H, Tian J, He W, Guo Z (2015). J Am Chem Soc.

[59] Nicholas S, Brown RB. Breast Cancer Res 2001;3: 323–327. 10.1186/bcr315 PMC138696 11597322

[60] Helen J, Knowles ALH. Breast Cancer Res 2001;3: 318–322. 10.1186/bcr314 PMC138695 11597321

[61] Feng W, Gao C, Liu W, Ren H, Wang C, Ge K, Li S, Zhou G, Li H, Wang S, Jia G, Li Z, Zhang J (2016). Chem Commun (Camb).

[62] Dzamukova MR, Naumenko EA, Lvov YM, Fakhrullin RF (2015). Sci Rep.

[63] Hu Q, Katti PS, Gu Z (2014). Nanoscale.

[64] de la Rica R, Aili D, Stevens MM (2012). Adv Drug Deliv Rev.

[65] Linderoth L, Peters GH, Madsen R, Andresen TL (2009). Angew Chem Int Ed Engl.

[66] Li H, Yu SS, Miteva M, Nelson CE, Werfel T, Giorgio TD, Duvall CL (2013). Adv Funct Mater.

[67] Park J, Yang J, Lim EK, Kim E, Choi J, Ryu JK, Kim NH, Suh JS, Yook JI, Huh YM, Haam S (2012). Angew Chem Int Ed Engl.

[68] Shi H, He X, Wang K, Wu X, Ye X, Guo Q, Tan W, Qing Z, Yang X, Zhou B (2011). Proc Natl Acad Sci U S A.

